# Zebrafish models of inherited retinal dystrophies

**DOI:** 10.20517/jtgg.2021.47

**Published:** 2022-02-08

**Authors:** Brian D. Perkins

**Affiliations:** 1 Department of Ophthalmic Research, Cole Eye Institute, Cleveland Clinic, OH 44195, USA.; 2 Department of Ophthalmology, Cleveland Clinic Lerner College of Medicine, Case Western Reserve University, OH 44195, USA.; 3 Department of Molecular Medicine, Cleveland Clinic Lerner College of Medicine, Case Western Reserve University, Cleveland, OH 44195, USA.

**Keywords:** Retina, photoreceptors, zebrafish, regeneration, degeneration, cilia

## Abstract

Inherited retinal degenerations (IRDs) cause permanent vision impairment or vision loss due to the death of rod and cone photoreceptors. Animal models of IRDs have been instrumental in providing knowledge of the pathological mechanisms that cause photoreceptor death and in developing successful approaches that could slow or prevent vision loss. Zebrafish models of IRDs represent an ideal model system to study IRDs in a cone-rich retina and to test strategies that exploit the natural ability to regenerate damaged neurons. This review highlights those zebrafish mutants and transgenic lines that exhibit adult-onset retinal degeneration and serve as models of retinitis pigmentosa, cone-rod dystrophy, and ciliopathies.

## INTRODUCTION

Inherited retinal degenerations (IRDs) comprise a large collection of diseases that progressively compromise vision and can lead to blindness due to the degeneration of rod and cone photoreceptors. IRDs exhibit genetic and clinical heterogeneity, with 280 unique disease genes identified to date^[1]^. The inheritance patterns of IRDs include autosomal dominant, autosomal recessive, and X-linked, although mutations in mitochondrial DNA have been shown to cause retinopathy. Diseases such as retinitis pigmentosa (RP), cone-rod dystrophy, Leber congenital amaurosis (LCA), and choroideremia affect the eye alone. RP is also associated with several syndromic and systemic conditions, where the disease impacts one or more other organs. These diseases can include Usher syndrome, in which RP is associated with hearing loss, Joubert syndrome (JBTS) and Bardet-Biedl syndrome (BBS), which are two ciliopathies with systemic effects, and Senior-Loken syndrome, which presents with RP and kidney disease. It was recently estimated that approximately 5.5 million people worldwide (~1 in 1380 people) are afflicted by IRDs^[2]^ and this has a significant cost on both individuals and society. The financial impact of IRDs associated with economic and wellbeing costs was estimated to range between $13–31 billion in the United States and between CAN$669–1638 billion in Canada for 2019^[3]^.

Until recently, no therapeutic options existed that could slow or reverse the disease progression of IRDs. The ongoing success in identify the genetic causes of IRDs has allowed strategies such as gene therapy and genome editing to show promise and early success^[4–6]^. While encouraging, gene augmentation and gene correction strategies still require that most therapies be specifically tailored to each genetic mutation or mutated exon, of which there are thousands. To circumvent these limitations, there has been considerable interest in strategies that promote tissue regeneration either through stem cell transplantation^[7,8]^ or regeneration from endogenous stem cell populations^[9,10]^. Although adult mammals show limited potential for endogenous retinal regeneration^[11,12]^, zebrafish readily regenerate their retina after injury^[13–15]^ and restore visual function^[16]^. In zebrafish, the Müller glia respond to injury by reprogramming and re-entering the cell cycle to produce multipotent neural progenitors^[15,17,18]^. There is now considerable interest in exploiting this regenerative capacity for possible therapies for the treatment of IRDs. As no single therapeutic strategy will be a “magic bullet” for IRDs, including regenerative medicine, it remains essential to test any approaches in animal models that recapitulate human disease.

Since being established as an animal model for developmental genetics in the 1970s^[19,20]^, zebrafish have become increasingly popular as a disease model, including as a model for IRDs^[21–28]^. Investigators initially used genetic screens of fish mutagenized by ethylnitrosourea (ENU) to identify dozens of mutants with defects in retinal structure and function^[29–33]^. Simple histological methods^[31,34,35]^ and visual behaviors, such as the optokinetic response (OKR)^[29,30]^, were highly successful at identifying novel mutant phenotypes in larval zebrafish and ultimately discovering factors important for protein trafficking^[36]^, cone phototransduction^[37,38]^, retinal development^[39,40]^, and many others. Interestingly, almost all of these mutations affects cones or cones and rods and the large majority were lethal within the first two weeks of age. Why would impaired vision result in lethality? Insight comes from the study of the *no optokinetic response f* (*nof*) mutant, which was identified in ongoing screens for mutants that lacked an OKR behavior^[38]^. The *nof* mutation introduced a stop codon in the alpha subunit of cone transducin, leading to complete loss of protein. The *nof* mutants were morphologically indistinguishable from their wild-type siblings and no evidence of retinal degeneration was observed. Nevertheless, the *nof* larvae did not survive beyond 12 dpf under standard rearing conditions. If the mutants were raised in a “soup” containing a 10-fold higher concentration of paramecium, a large number survived to adulthood. The conclusion was that loss of cone function impaired the ability of larvae to effectively hunt paramecia using visual cues and feed during the first few weeks of life. In other words, severe visual impairment leads to starvation under standard laboratory rearing conditions. A notable exception was the early search for dominant forms of retinal degeneration in zebrafish by Li and Dowling^[33]^. In this study, the investigators used the “escape response” behavior assay to screen for mutants. Zebrafish swim in circles when placed in a small, circular container but will rapidly turn and swim in the opposite direction when challenged. F1 animals (i.e., heterozygotes) derived from ENU mutagenized founders were dark adapted and screened for elevated visual thresholds to identify mutants. The *night blindness a* (*nba*) mutant exhibited elevated visual thresholds and progressive degeneration of rods and cone^[33]^. The identity of the *nba* gene remains unknown, but this was the first reported mutant with adult-onset retinal degeneration in zebrafish. As ENU creates numerous lesions throughout the genome, it is important that all ENU-induced mutants be outcrossed for several generations to ensure the observed phenotypes reflect the mutation of a single locus. Genome editing technologies such as CRISPR/Cas9 now permit investigators to generate zebrafish with mutations in genes orthologous to human disease genes^[41,42]^ and other genes of interest. While most zebrafish mutants have been investigated primarily at the larval stage, a number of zebrafish mutants are viable into adulthood and show progressive photoreceptor degeneration. In contrast to the regeneration observed following acute injury, these mutants do not initiate a robust regeneration response to disease. The reasons for this difference remain unclear. The purpose of this review is to examine those zebrafish mutants that are adult-viable and exhibit progressive retinal degeneration. Studies of zebrafish mutants with photoreceptor dysfunction during larval stages have provided valuable information regarding photoreceptor biology; however, in order to harness the capacity for regeneration in a diseased retina, it will be critical to investigate zebrafish models that mimic the pathology and progressive degeneration observed in human IRDs. Several recent reviews provide excellent discussions of regeneration^[43–46]^ and of zebrafish mutants with retinal degeneration^[22,27,47–49]^.

## ZEBRAFISH MODELS OF RETINAL DEGENERATION

Several zebrafish models of retinal dystrophy exist. In some cases, retinal degeneration is induced by damage caused by cytotoxic compounds^[16,50,51]^, acute light exposure^[14]^, or mechanical injury^[15]^ for the purposes of studying regeneration. This review will focus on genetic models with progressive retinal degeneration in adults and will include both transgenic lines and zebrafish mutants with mutations in genes that cause retinal dystrophy in humans.

### Zebrafish mutant models of progressive retinal dystrophy

#### Abelson-helper integration site-1

Mutations in the *Abelson-helper integration site-1* (*AHI1*) gene result in JBTS^[52,53]^. JTBS is an autosomal recessive ciliopathy noted for a characteristic brain-stem abnormality known as the “molar tooth” sign, as well as retinal dystrophy. The *AHI1* gene encodes a protein component of the ciliary transition zone (TZ) that functions as a diffusion barrier between the photoreceptor inner segment and outer segment^[54,55]^. The zebrafish *ahi1*^*lri46*^ mutant exhibited disorganized cone outer segments during larval stages but visual function as assessed by OKR assays was not impaired at 5 days post fertilization^[41]^. More than 95% of zebrafish *ahi1* mutants died before 3 months post fertilization (mpf). Among surviving animals, cone photoreceptor structure was normal at 3 mpf but rapidly degenerated by 5 mpf. Rod photoreceptors exhibited rhodopsin mislocalization but a net loss of rods was not observed, suggesting that rods were unhealthy but degeneration was limited.

#### Aryl hydrocarbon receptor interacting protein-like 1

Mutations in the aryl hydrocarbon receptor interacting protein-like 1 (*AIPL1*) gene result in LCA, although individuals heterozygous for a 12-bp deletion were diagnosed with either a dominant form of juvenile retinitis pigmentosa or autosomal dominant cone-rod dystrophy^[56]^. *AIPL1* is expressed in exclusively in rod photoreceptors of both the peripheral and central retina in humans^[57]^. Aipl1 protein functions as part of an HSP90 chaperone complex^[58]^ that facilitates maturation of farnesylated proteins such as the cGMP phosphodiesterase 6 subunits α and β^[59,60]^. The zebrafish *gold rush* (*gosh*) mutant was identified in a large-scale mutagenesis screen for ENU-induced recessive mutations that disrupt visual behavior in zebrafish larvae^[32]^. Visual function was completely absent in *gosh* mutants and cone photoreceptors were disorganized with shorter outer segments at 7 dpf. By 4 wpf, the ONL was thinner and green cones were absent. Cones were almost completely absent by 12 wpf and the number of neurons in all layers of the retina were decreased. Rods continued to be maintained, although rhodopsin staining was reduced compared to wild-type siblings. Interestingly, proliferation of rod precursors in the ONL of *gosh* mutants was observed beginning at 5 wpf^[61]^. There was also a small increase in the number of proliferating Müller glia in the INL of *gosh* mutants. Subsequent genetic mapping of the *gosh* locus identified an *aipl1* gene as a candidate for the *gosh* mutant. Sequencing of this gene uncovered a nonsense mutation. The zebrafish genome contain two *aipl1* orthologs, *aipl1a* and *aipl1b*. The *aipl1a* gene is more similar to human *AIPL1*. The *gosh* mutant was caused by a mutation in the *aipl1b* gene. Expression of *aipl1b* was restricted to cones, whereas *aipl1a* was expressed in rods and likely UV cones. The cone-specific expression of *aipl1b* likely explains why cones degenerated in *gosh* mutants. Cones degenerated rapidly in *gosh* mutants but rods also begin degenerating by 3 weeks post fertilization, indicating that *aipl1b* is necessary for both cone and rod survival^[61]^. A follow-up study found that the rapid degeneration of cones triggered a modest proliferation of Müller glia and rod degeneration resulted in proliferation of rod precursors. Within the INL, the proliferating Müller glia expressed Sox2 and the neural progenitors expressed Pax6. Individual Pax6+ cells appeared to migrate to the ONL. Given that the proliferating Müller glia did not result in cone regeneration, it is possible that the Pax6+ neural precursors were rod progenitors en route to the ONL to provide additional rod precursors and attempt to regenerate rods^[62]^.

#### BBS2

BBS is clinically and genetically heterogeneous autosomal recessive disorder characterized by retinal degeneration, obesity, renal dysfunction, polydactyly, and mental retardation^[63,64]^. To date, mutations in 22 genes have been shown to cause BBS^[65]^. *BBS2* encodes a core protein of the BBSome^[66]^, which is an octomeric protein complex required for protein trafficking through the photoreceptor cilium^[67,68]^. The *bbs2*^*lri82*^ mutant was generated by CRISPR/Cas9 mutagenesis and exhibited impaired OKR responses and shortened cone outer segments at 5 dpf^[69]^. The *bbs2* mutants lived into adulthood, but were smaller and exhibited spinal curvatures that are consistent with ciliopathy phenotypes in zebrafish^[70]^. In adult *bbs2* mutants, cone photoreceptors degenerated and rods exhibiting rhodopsin mislocalization. A significant increase in proliferating cells within the outer nuclear layer of adult *bbs2* mutants suggested that rods were being regenerated from rod precursors. No proliferation of Müller glia was observed in *bbs2* mutants, indicating that the regeneration typically seen following acute injury did not occur. The lack of Müller glia proliferation was not due to the *bbs2* mutation, as acute light injury was capable of triggering a regeneration response in both *bbs2* mutants and wild-type siblings.

#### Centrosomal protein 290-kD

The *centrosomal protein 290-kD* gene (*CEP290*) encodes a large, multidomain coiled-coil protein that localizes to the ciliary TZ and to the centrosome/basal body in motile and non-motile cilia^[71–73]^. As a component of the TZ, Cep290 regulates protein trafficking through the cilium^[74]^ and facilitates ciliary assembly^[75,76]^. In humans, more than 130 mutations in *CEP290* have been shown to cause a variety of ciliopathy disorders, including JBTS, BBS, Senior-Loken syndrome, Meckle syndrome, and nephronophthisis^[77,78]^, with retinal dystrophy being a common symptom. Mutations in *CEP290* can also result in non-syndromic retinal degenerations such as early-onset LCA^[79]^ and late-onset RP^[80]^. Despite several years of intense study on *CEP290* genetics in humans and in animal models, no consensus exists to explain CEP290 pleiotropy or to establish genotype-phenotype correlations. Three distinct zebrafish *cep290* mutant lines have been described in the literature. The *cep290*^*fh297*^ allele is a nonsense point mutation (p.Gln1217X) that was induced by ethynitrosourea (ENU)^[81]^. The *cep290*^*fh297*^ mutant had normal visual function by OKR assays and normal photoreceptor structure at 5 dpf^[81]^. The *cep290*^*fh378*^ mutant allele was made using TALEN genome editing and resulted in a 2 bp deletion that produces a downstream stop codon after amino acid 99^[82]^. No retinal phenotype was described for *cep290*^*f378*^. The *cep290*^*fb208*^ mutant allele is a 10 bp deletion in exon 16 generated by CRISPR/Cas9 mutagenesis and results in a premature stop codon^[83]^. Cardenas-Rodriquez and colleagues generated maternal-zygotic (MZ) *cep290*^*fb208*^ mutants, which lack maternally-derived mRNA and protein in the yolk of zebrafish embryos and larvae, to exclude the possibility that the presence of maternal Cep290 protein could mask phenotypes during larval stages^[83]^. *MZcep290*^*fb208*^ mutants also exhibited normal vision as assessed by electroretinograms but did show mild disorganization of outer segments and an accumulation of vesicular material at the base of the outer segments^[83]^. In adults, both *cep290*^*fh297*^ and *cep290*^*fb203*^ mutants exhibited progressive cone photoreceptor degeneration, with the *cep290*^*fh297*^ mutant losing approximately 40% of cones by 6 mpf and 80% of cones by 12 mpf. Rhodopsin mislocalized to the inner segments of rod photoreceptors. The proliferation of rod precursors in the ONL suggested that rods degenerated in the *cep290*^*fh297*^ mutant^[81]^. Similar to what was observed in the *bbs2* mutant, Müller glia did not proliferate in the *cep290*^*fh297*^ mutants. Analysis of Müller cell proliferation or rod degeneration was not performed for the *MZcep290*^*fb208*^ mutants.

#### Ceramide kinase-like

The *ceramide kinase-like* (*CERKL*) gene encodes a 532 amino acid polypeptide that contains a pleckstrin homology (PH) and a diacylglycerol kinase (DAGK) domain^[84]^. *CERKL* is a member of the ceramide kinase protein family, which converts ceramide to ceramide-1-phosphate, but CERKL has not yet been shown to possess enzymatic activity^[85,86]^. *CERKL* is expressed throughout the retina, as well as brain, lung, liver, kidney, and pancreas^[84]^. Despite this widespread expression pattern, mutations in *CERKL* have only been associated with nonsyndromic RP^[84]^ and with cone-rod dystrophy^[87]^. A *cerkl* knockout zebrafish (*cerkl*^*hzu3*^) was generated by TALEN technology and resulted in a 7 bp deletion that caused a premature nonsense mutation. Visual impairment was detected in 7 dpf *cerkl* larvae by ERG analysis^[88]^. Thinning of the ONL was observed as early as 2 mpf, with continued thinning at 4 mpf and a significant loss of photoreceptors by 12 mpf. Immunohistochemical and western blotting analyses indicated that rod photoreceptor degeneration preceded the degeneration of cones, suggesting that the *cerkl* mutant represents an ideal model for RP. Cell proliferation was not assessed for the *cerkl* mutant, so it remains unclear if the rod precursors or Müller glia attempt to regenerate photoreceptors. The mechanisms underlying photoreceptor degeneration due to loss of CERKL function remain unclear and future studies of the *cerkl* zebrafish mutant may provide unique insight.

#### Eyes shut

The *eyes shut* (*EYS*) gene encodes a large extracellular matrix protein of 3165 amino acids that is the homolog of the *Drosophila eyes shut* gene. Mutations in *EYS* are a major cause of autosomal recessive RP in humans across the world and account for the most prevalent form of RP in Japanese populations^[89,90]^. *EYS* is highly expressed in the human retina but expression is absent in the retinas of mouse, rat, and cattle^[91]^, thus limiting the study of EYS function in the retina. To address this limitation three groups independently generated mutations in the zebrafish *eys* gene. Yu *et al*.^[92]^ used CRISPR/Cas9 to generate multiple mutants with truncating nonsense alleles in exon 2 of the *eys* gene. Three alleles, *eys*^*sny10*^, *eys*^*sny13*^, and *eys*^*sny14*^, had identical phenotypes of cone degeneration beginning between 4–6 mpf and slower rod degeneration that was apparent by 14 mpf. Using antibodies specific for EYS, the group also reported that EYS protein concentration near the connecting cilium and transition zone of the photoreceptors in both zebrafish and primate retinas. Using TALEN technology, Lu *et al*.^[93]^ independently generated several truncating nonsense alleles in exon 47 of the zebrafish *eys* gene. ERG analysis at 10 dpf revealed a significant decrease in b-wave amplitude in *eys* mutants, indicating visual impairment. In these mutants, a decrease in ONL thickness was noticed as early as 2 mpf, with more than 60% reduction by 16 mpf. Interestingly, cone subtypes differed in the rate of degeneration. Lu *et al*.^[93]^ reported that the number of red and UV cones decreased significantly by 4 mpf, while green and blue cones were only partially reduced by 8 mpf. It is unclear why zebrafish with mutations in exon 47 would exhibit more rapid degeneration of cones and the selective loss of red and UV cones, when compared to zebrafish with mutations in exon 2. More recently, Messchaert *et al*.^[94]^ used CRISPR/Cas9 technology to generate a 5-bp deletion in exon 20 of *eys*. Immunohistochemistry with anti-EYS antibodies on retinas from the *eys*^*rmc101*^ mutant showed a complete loss of protein, indicating this was a null allele. In 5 dpf larvae, the *eys*^*rmc101*^ mutant was reported to have shorter and more disorganized photoreceptor outer segments than wild-type siblings, but no quantification was provided. By 2 mpf a significant thinning of the INL and ONL was observed. Rhodopsin and the alpha subunit of cone transducin were mislocalized in rods and cones, respectively by 5 mpf. The authors also explored Müller glia morphology and did not observed evidence of gliosis or Müller glia proliferation. Of note, however, was that *eys*^*rmc101*^ mutants had normal OKR behavior but a decrease in the ERG b-wave, which strongly suggested an early defect in visual function as early as 5 dpf. All three groups reported that *eys* mutants exhibited photoreceptor degeneration that starts by 2 mpf and progresses with age. Additional investigation with these mutants will help reveal the cellular function of EYS in photoreceptors and explain the mechanisms leading to photoreceptor death.

#### Photoreceptor cilium actin regulator

Two groups independently identified mutations in the *centrosome 2 open reading frame 71* (*C2ORF71*) gene that result in non-syndromic autosomal recessive RP in families of various national origins^[95,96]^. *C2ORF71* is exclusively expressed in the retina^[95]^. Recent work demonstrated that the protein encoded by *C2ORF71* interacts with several proteins associated with centrioles, microtubules, and factors that regulate actin filament assembly^[97]^. Based on these findings, Corral-Serrano *et al*.^[97]^ proposed that *C2ORF71* be renamed *PCARE* for photoreceptor cilium actin regulator. The zebrafish genome contains two *pcare* paralogs, *pcare1* and *pcare2*^[98]^. By searching for synteny between the chromosomal region of the human *PCARE* gene and the zebrafish genomes, the *pcare1* gene was considered to be the ortholog of the human gene. A zebrafish *pcare1* mutant line was generated by CRISPR/Cas9 mutagenesis and the 29 bp deletion was predicted to result in a truncating nonsense allele at amino acid 16^[98]^. Mutants exhibited disrupted actin assembly and disorganized photoreceptor structure as early as 5 dpf. The *pcare1* mutants were viable and at 6 mpf, the rod outer segments were highly disorganized or missing and cone outer segments were significantly shorter than wild-type siblings^[98]^. This is consistent with the RP phenotype in humans. The rapid degeneration of rods seen in *pcare1* mutants would suggest that proliferation of Müller glia or rod precursors may occur, although this was not investigated. Future studies of the *pcare1* mutant could explore whether a regenerative response follows the degeneration of photoreceptors, while functional studies of *pcare2* may uncover a similar role in photoreceptor maintenance.

#### Phosphodiesterase 6

Photoreceptors respond to light by activating a G-protein signaling pathway known as the phototransduction cascade^[99,100]^. Upon absorption of a photon of light, rhodopsin or cone opsins activate many molecules of the heterotrimeric G-protein transducin. The GTP-bound α-subunit of transducin (Gα_t_) activates its effector enzyme, cGMP phosphodiesterase 6 (PDE6). PDE6 hydrolyzes cGMP, resulting in a decrease in cGMP concentration and the closure of cGMP-gated channels in the outer segment plasma membrane. PDE6 is a multimeric enzyme. The rod-specific PDE6 is a heterotetramer, consisting of a α-subunit, β-subunit, and two γ-subunits. The cone PDE6 consists of two cone-specific α’-subunits and two cone-specific γ-subunits. The cone-specific α’-subunits are encoded by the *PDE6C* gene^[101]^, Mutations in the rod-specific *PDE6A* gene cause RP^[102]^ while mutations in *PDE6C* result in cone dystrophy and achromatopsia^[103,104]^. Nishiwaki *et al*.^[105]^ conducted a genetic screen for zebrafish with defects in visual behavior and identified several mutants, including *eclipse* (*els*). The *els* mutant exhibited irregular photoreceptor structure and a complete absence of the ERG b-wave. Subsequent mapping and cloning of the *eclipse* locus identified a missense mutation in the *pde6a’* (*pde6c*) gene that resulted in a Met175Arg amino acid substitution. They found that *pde6c* was expressed in cones and that cones degenerated rapidly. Despite the lack of functional cone vision, the mutants survived into adulthood. Few cones remained at 3 wpf and although rod morphology was compromised at 3 wpf, the rods remained functional. By 6 mpf, rods appeared normal but cones were absent from the central retina^[106]^. As early at 5 wpf, the number of proliferating cells (BrdU+) was increased in both the INL and ONL of the *pde6c* mutants. At 5 wpf, however, the retina continues to rapidly expand and add new rods into the cone mosaic as the fish grows. It is unclear if the proliferation observed in the *pde6c* mutants reflects an attempt to regenerate dying rods, the expansion of rod numbers as the fish grows, or a combination of both. The significant increase in proliferating cells within the INL, however, may suggest a regenerative response due to the rapid degeneration of cones. Recently, a mutation in the rod-specific *pde6a* gene was reported^[107]^. This mutant had been generated by ENU mutagenesis and identified at the European Zebrafish Resource Centre. The mutation introduced a stop codon that produced a truncated protein at glutamine 70 (*pde6a*^*Q70X*^). Disruption of *pde6a* did not result in OKR defects at 5 dpf, as the OKR is a cone-driven response^[29]^. The visual motor response (VMR) assay measures the locomotor activity of larvae to rapid light-dark changes^[108]^. In VMR assays, the *pde6a* mutant had reduced VMR responses, suggesting impaired ability to detect light. Mutation of *pde6a* resulted in a reduction in rod size at 5 dpf and an almost complete absence of rods by 21 dpf. Cone numbers were reduced slightly at 21 dpf, but morphology appeared normal^[107]^. No results were reported for later ages. Future studies will be needed to determine the long-term effect of *pde6a* loss on cones and whether the proliferation of cells in the INL of *pde6c* mutants can be enhanced to potentially regenerate cones.

#### RP2

X-linked retinitis pigmentosa (XLRP) can present as an aggressive and early form of RP that can occur within the first 4 years of life^[109]^. Mutations in the *RP2* gene were found to cause XLRP and these account for approximately 16% of all XLRP cases^[110]^. *RP2* encodes a 350-amino acid protein that stimulates the GTPase activity of tubulin and also interacts with ADP-ribosylation factor-like-3 (ARL3) to stimulate its GTPase activity^[111,112]^. RP2 binds membranes and localizes to the cilium and photoreceptor membrane^[111,113]^. Based on these observations, RP2 has been proposed to facilitate vesicular trafficking of proteins from the Golgi to the connecting cilium^[113]^. The zebrafish *rp2* knockout line was generated using TALEN genome editing^[114]^. The *rp2* mutants completely lack Rp2 protein and show mild visual impairment at 7 dpf with a 30% reduction in the scotopic ERG b-wave compared to wild-type controls. Although mutants exhibited reduced visual function, no morphological differences appeared until 4 mpf, when rod outer segments were approximately 20% shorter. By 7 mpf, both rods and cones were shorter, consistent with a RP-like phenotype. Ultrastructural analysis of 10 mpf *rp2* mutants revealed that the rod outer segments were almost completely missing, while the remaining outer segments were disorganized. While the phenotype of the *rp2* mutant follows a progressive rod-cone dystrophy consistent with RP, it is worth noting that the rate of degeneration is quite slow compared to humans with *RP2* mutations. The *rp2* mutant may serve as a useful model to explore the mechanisms of photoreceptor degeneration and regeneration.

#### Retinitis pigmentosa GTPase regulator interacting protein 1

The *retinitis pigmentosa GTPase regulator interacting protein 1* (*RPGRIP1*) gene encodes a 1259 amino acid protein with several alternatively spliced isoforms and was first identified as a binding partner to RPGR^[115,116]^. Mutations in *RPGR* are responsible for the majority of cases of XLRP, while mutations in *RPGRIP1* result in LCA^[117,118]^ and cone-rod dystrophy^[119]^. *RPGRIP1* is highly expressed in the retina, with weaker expression only found in the testis^[116]^. RPGRIP1 protein colocalizes with RPGR at the photoreceptor connecting cilium^[120]^, while some isoforms localize to the outer segments and other subcellular structures, including the nucleus^[121]^. RPGRIP1 is proposed to anchor RPGR at the connecting cilium where they likely function within the ciliary transition zone to regulate protein trafficking^[122]^. A zebrafish *rpgrip1* mutant was made by ENU mutagenesis^[123]^. The zebrafish *rpgrip1* contains 1342 amino acids and the mutation introduced a stop codon at amino acid 736 (Q736X). At 7 dpf, rod outer segments failed to form in *rpgrip1* mutants, although cones were unaffected and the disk membranes appeared normal. Rhodopsin was mislocalized throughout the inner segments of the *rpgrip1* mutant rods. Rods degenerated rapidly and only a few rod nuclei were present by 3 mpf. By 6 mpf, cone degeneration was apparent. Rod degeneration preceded the loss of cones, which is consistent with an RP model with rod-cone dystrophy rather than the cone-rod dystrophy seen in humans. By 13 mpf, both rods and cones had degenerated and few photoreceptor nuclei could be found within the presumptive ONL. Cell proliferation was not reported for the *rpgrip1* mutant so future studies may explore whether rod precursors attempt to regenerate dying rods and whether the rapid degeneration triggers a response from Müller cells.

#### RHODOPSIN

Rhodopsin is the visual pigment of rod photoreceptors that absorbs photons of light to mediate vision. Rhodopsin is a proto-typical G-protein coupled receptor that binds the light-absorbing chromophore 11-cis-retinal within the transmembrane region of the protein^[124]^. Mutations in the *RHODOPSIN* (*RHO*) gene are responsible for approximately 18%–26% of all adRP cases, which is far more than any other gene^[125–127]^. Zebrafish possess two genes that encode rhodopsin, the *rh1-1* and *rh1-2* genes^[128,129]^. The protein product of the *rh1-1* gene shares strong homology with other vertebrate RHO proteins and the pattern of expression is consistent with function as the rod opsin gene. Multiple mutations in the zebrafish *rhodopsin* gene (*rh1-1*) were generated by CRISPR/Cas9 mutagenesis^[42]^. The *rho*^*fl6*^ allele encoded an N-terminal nonsense mutation at amino acid 17 (T17*), which resulted in rod degeneration in homozygous animals as early as 5 dpf. Heterozygous animals for the *rho*^*fl6*^ allele did not exhibit a phenotype, suggesting this mutation caused recessive rod degeneration. Two other N-terminal mutations, the *rho*^*fl7*^ and *rho*^*fl10*^ alleles, encoded in-frame deletions that disrupted a highly conserved glycosylation sequence. Heterozygous larvae of both the *rho*^*fl7*^ and *rho*^*fl10*^ alleles exhibited rapid rod degeneration by 6 dpf, consistent with a dominant rod degeneration. The *rho*^*fl8*^ allele encodes an in-frame deletion in the C-terminus of the protein, while the *rho*^*fl9*^ allele results in a nonsense mutation at amino acid 347 (S347*). Heterozygous larvae of both alleles show loss of rods in the central retina. In adults, few rod photoreceptors were present and the rod outer segments were missing in heterozygous animals. These new zebrafish mutants will serve as useful models of both adRP while the *rho*^*fl6*^ allele could serve as model for autosomal recessive RP. Interestingly, the zebrafish cone photoreceptors were unaffected by the loss of rods. This differs from humans with RP-causing mutations in *RHO*, where rod degeneration results in the indirect death of cones. Future work may uncover novel mechanisms that permit cone survival in the absence of rods and reveal potential targets for therapies to preserve cones in patients with RP.

### Transgenic zebrafish models of retinal degeneration

#### Tg(Xla.Rho:GAP-CFP)q13Tg

The first report of a model with rod-specific degeneration described a transgenic line of zebrafish that expresses a membrane-targeted cyan fluorescent protein (mCFP) using a 5.5 kb promoter sequence from the *Xenopus laevis* rhodopsin gene^[130,131]^. The mCFP protein included an N-terminal palmitoylation signal sequences from neuromodulin (GAP-43), which targets proteins to the plasma membrane^[132]^. This was originally named the *Tg(XOPS:mCFP)* transgenic line^[130]^. The Fadool lab had previously generated a transgenic line of zebrafish that expressed eGFP from the *Xenopus* rhodopsin promoter^[133]^. This *Tg(XOPS:eGFP)* line specifically labeled rods with eGFP and did not result in any deleterious effects on rod photoreceptors^[133]^. In the *Tg(XOPS:mCFP)* line, however, rods exhibited an abnormal morphology shortly after the onset of transgene expression and most rods were missing by 5 dpf. Expression of the mCFP caused rhodopsin mislocalization to the inner segments and rapid degeneration. It is possible that the genomic integration sites of the *Tg(XOPS:mCFP)* transgene could have affected rod function, although this is considered unlikely. Zebrafish carrying the *Tg(XOPS:mCFP)* transgene survived into adulthood but lacked almost all rod photoreceptors. Cone photoreceptors survived into adulthood and cone morphology, and the cone mosaic appeared normal in the transgenic animals. This was the first indication that rod degeneration did not result in the secondary death of cones in zebrafish. The authors also noted an increase in the proliferation of rod precursor cells within the ONL of *Tg(XOPS:mCFP)* adults. The *Tg(XOPS:mCFP)* line has been instrumental in identifying the separate genetic pathways for rod and cone regeneration^[134]^, identifying genes important for rod regeneration^[135]^, and to demonstrate that an 11-cis-retinyl ester cycle is critical to maintain cone vision^[136]^.

#### Inducible rod death: Tg(rho:YFP-ntr)gmc500Tg, Tg(rho:Eco.NfsB-EGFP)nt19Tg, and Tg(rho:Eco.NfsB-EGFP)nt20Tg

To study the effects of selective photoreceptor death in a controlled fashion, two groups independently generated transgenic lines that show rod-specific expression of the *E. coli* nitroreductase enzyme fused to a fluorescent reporter gene^[137,138]^. The nitroreductase enzyme (NTR, *nfsB* gene) reduces nitroimidazole prodrugs, such as metronidazole (MTZ), into cytotoxic metabolites that crosslink DNA and cause rapid death. When transgenic zebrafish are exposed to MTZ, any cells expressing NTR are selectively and specifically eliminated. Montgomery *et al*.^[138]^ first published the *Tg(rho:Eco.NfsB-eGFP)nt19Tg* and *Tg(rho:Eco.NfsB-eGFP)nt20Tg* alleles to investigate how rod death induces regeneration. Both lines utilize a 1.2 kb fragment of the zebrafish rhodopsin promoter to express a NTR-eGFP fusion protein specifically in rods. In the absence of MTZ, rods express NTR-eGFP and GFP fluorescence is visible in the rod outer segments and inner segments. Upon exposure to 10 mM MTZ for 24 h, all the rods in the retinas of the *Tg(rho:Eco.NfsB-eGFP)nt19Tg* line rapidly degenerated within 48 h. Despite carrying the identical transgene, the *Tg(rho:Eco.NfsB-eGFP)nt20Tg* transgenic line displayed NTR-eGFP expression in only a subset of rods. The genomic integration sites of the transgene most certainly differed between the *nt19Tg* and *nt20Tg* alleles and this may have influenced the expression patterns. When the *Tg(rho:Eco.NfsB-eGFP)nt20Tg* transgenic fish were exposed to MTZ for 24 h, only those rods expressing NTR were destroyed and the non-expressing rods survived. The difference in damage between the two lines allowed the authors to compare how the retina responds to the loss of a subset of rods compared to the acute ablation of all rods photoreceptors. When a subset of rods was destroyed in the *Tg(rho:Eco.NfsB-eGFP)nt20Tg* transgenic line, rod precursor proliferation in the ONL was increased. The acute loss of all rods following MTZ treatment in *Tg(rho:Eco.NfsB-eGFP)nt19Tg* transgenic animals triggered a regenerative response by Müller glia^[138]^. Independently, Ariga *et al*.^[139]^ and Walker *et al*.^[140]^ generated the *Tg(rho:YFP-ntr)gmc500* transgenic line, which utilizes a 3.7 kb fragment of the zebrafish rhodopsin promoter to drive a *YFP-nfsB* transgene. The *Tg(rho:YFP-ntr)gmc500* transgene was also expressed in all rod photoreceptors, indicating that the 3.7 kb promoter and the 1.2 kb promoter both contain proper elements for rod-specific expression. Exposing *Tg(rho:YFP-ntr)gmc500* transgenic larvae to MTZ resulted in rod-specific death within 48 h. Time-lapse *in vivo* imaging of demonstrated that peripheral macrophages and resident microglia rapidly transitioned to an amoeboid morphology following rod death in 5–7 dpf larvae^[137]^. No studies were performed on adult animals. The use of the MTZ/NTR system enables investigators to selectively ablate rods in an inducible manner. The ability to trigger acute damage to a specific cell type at a pre-determined time allows investigators to compare the regenerative response between acute injury and chronic degeneration models.

#### Tg(rho:Mmu.Rho_P23H-FLAG)uth4Tg

Transgenic models of adRP that express mutant forms of rhodopsin in rod photoreceptors have been made in mice^[141–143]^, rats^[144]^, and frogs^[145–148]^. These models exhibit progressive rod degeneration and have proven invaluable toward understanding the mechanisms of degeneration caused by different pathogenic mutations in the rhodopsin gene. To determine if a zebrafish model of RP could be utilized to study the molecular signals stimulating regeneration, a construct was made that contained a 1.8 kb fragment of the zebrafish rhodopsin promoter to drive expression of a mouse rhodopsin carrying the P23H mutation fused to a C-terminal Flag tag^[149]^. The P23H mutation was chosen as it is the most frequent rhodopsin mutation to cause adRP and was the first mutation identified in humans^[150]^. The resulting stable transgenic line, *Tg(rho:Mmu.Rho_P23H-FLAG)uth4Tg* expressed the P23H-rhodopsin-FLAG beginning at 3 dpf. The mutant rhodopsin protein was mislocalized throughout the cell body and synapses in larval animals. Rod outer segments were shorter in larval stages but cones appeared normal. In adult transgenic animals, very few rods were observed and the P23H-rhodopsin-FLAG protein was significantly mislocalized in the remaining rods. Endogenous wild-type rhodopsin was also mislocalized, indicating that expression of the P23H-rhodopsin had deleterious effects on trafficking of outer segment proteins. Adult transgenic animals had a 3-fold reduction in the number of nuclei in the ONL, indicating degeneration of rods. Interestingly, cone inner segments and outer segments were shorter in transgenic animals compared to adults, although the total number of Zpr1+ cones was unchanged. These results suggested that transgenic expression of a P23H-rhodopsin mutant causes secondary damage to cones. This differs from the rhodopsin (*rh1-1*) knockout zebrafish mutants, which had normal cone morphology^[42]^. At both 4 mpf and 6 mpf, it was noticed that the number of proliferating cells (PCNA+) was considerably higher in the ONL of transgenic animals compared to wild-type. There was no signs of proliferation within the INL. Cell proliferation was confirmed by BrdU labeling and many of the BrdU+ cells also expressed rhodopsin, indicating that the newly post-mitotic cells were rods. Together, these results demonstrate that rod degeneration caused by expression of a P23H-rhodopsin transgene can initiate a regeneration response from rod precursors but not from Müller glia.

## PERSPECTIVES

The mutants and transgenic models described herein represent a variety of human retinal dystrophy conditions, ranging from ciliopathies to RP to cone-rod dystrophy. As zebrafish also possesses the capacity to regenerate, it remains unclear why the response to progressive degeneration differs so greatly from acute damage. Inflammation is believed to be critical for the initial reprogramming of Müller glia^[151]^, but excess inflammation compromises survival of regenerated photoreceptors^[152]^. Whether chronic inflammation may limit Müller glia reprogramming remains unanswered. It is also known that Notch signaling suppresses regeneration^[153,154]^. Following acute injury, expression of *notch3* is significantly downregulated prior to Müller glia proliferation. Understanding potential differences in the transcriptional responses of Müller glia^[155]^ and microglia^[156]^ to disease and injury will hopefully help answer why zebrafish do not regenerate in these IRD models.

## CONCLUSION

The zebrafish represents an ideal genetic model to study the pathology of retinal degeneration in a cone-rich retina. The development of new genetic tools for genome editing allow investigators to target candidate genes of interest. Indeed, many of the models described herein were generated by genome editing technologies. The power of unbiased forward genetic screens thrust zebrafish into the scientific mainstream by uncovering genes essential for vertebrate development and function. In the future, more sophisticated or sensitive forward genetic screening approaches could be utilized to identify adult-onset phenotypes in a space- and cost-efficient manner that will reveal even more genes and pathways critical for the health and survival of photoreceptors.
